# Deep Learning Image-Based Fusion Approach for Identifying Multiple Apparent Diseases in Concrete Structure

**DOI:** 10.3390/s25216796

**Published:** 2025-11-06

**Authors:** Yongsheng Tang, Yaomin Wei, Lengfeng Qian, Long Liu

**Affiliations:** 1 College of Civil and Transportation Engineering, Hohai University, Nanjing 210098, China; 2 Zhejiang Second Construction Group Co., Ltd., Ningbo 315000, China

**Keywords:** multi-disease identification, fusion network, deep learning, YOLO, UNet

## Abstract

Addressing the key pain points in detecting typical apparent diseases of concrete structures, where standalone object detection fails to achieve pixel-level quantification and standalone semantic segmentation, is inefficient. Therefore, a deep learning image-based fusion approach is proposed to identify the typical visible diseases in concrete structures, namely crack, spalling, water leakage, and seam deformation. To implement the approach, a deep learning fusion network is developed with the YOLO and UNet models to identify multiple apparent diseases rapidly. In the fusion network, the YOLO model is used to filter the images containing the visible diseases from all the images in the first stage. Then, the UNet model is used to extract the pixels containing diseases from the selected images. Lastly, analysis methods are proposed to quantify the diseases based on the segmented pixels, such as length, width, and area. In this paper, a dataset of 1488 images with the above diseases from a field inspection was used to train the deep learning fusion network. The training results demonstrated the robustness of the fusion network in identifying and segmenting diseases with a mean average precision of 0.72 and a Dice score of 0.82. Experiments were finally conducted on concrete slabs with simulated diseases for additional validation. The results indicated that the proposed fusion network could identify the diseases approximately 50% faster than the UNet model only. The quantification precision was found to be satisfactory, with relative errors below 11.07% for the area of water leakage, below 5% for the length and area of cracks, and below 6% for the width of seams.

## 1. Introduction

During service, tunnel lining concrete structures are susceptible to numerous diseases, including cracks, water leakage, spalling, and seam deformation. These diseases can compromise the functional performance of the tunnel and even lead to serious safety incidents. Consequently, timely and accurate detection and assessment of these issues are crucial [1,2].

The current methods for detecting these visible diseases primarily rely on manual visual inspection or the use of mechanical equipment. These approaches are time-consuming, labor-intensive, and often inefficient. Moreover, the subjective nature of manual inspections and the professional expertise of the inspectors can substantially influence the quality of the assessments [3,4]. Thus, traditional monitoring techniques fall short of meeting the demand for rapid, precise detection. In the field of digital image processing, several edge detection methods have been applied, such as the Scharr and Canny algorithms, which focus on threshold segmentation of image pixels to delineate the characteristic contours of targets. Notable research includes the work at early by Liu et al., utilizing digital cameras and image processing to measure crack widths and water leakage areas in tunnel linings [5]. However, the complexities of real-world environments, such as low lighting, dirty concrete surfaces, and low contrast of diseases, require manual adjustments of models and camera settings for optimal edge extraction. Moreover, existing models are limited to detecting a single type of disease, which hinders the simultaneous identification and analysis of multiple diseases. These challenges underscore the limitations of solely relying on traditional digital image processing for the automated, intelligent detection of structural diseases in tunnel linings [6,7].

Deep learning, a subset of machine learning that automates feature learning and processing, has achieved remarkable advances in computer vision. As its applications mature, this technology has been widely applied to the detection of concrete structural diseases globally. In 2019, Wu et al. introduced a Mask R-CNN (Region Convolutional Neural Network) model for leakage detection utilizing transfer learning from small-scale datasets, which considerably enhanced detection capabilities compared with traditional methods [8]. In subsequent years, innovative models continued to evolve. Man et al. in 2022 proposed two ResNet (Residual Network) models of varying depths for tunnel damage detection, demonstrating that deeper network architectures could identify tunnel damages more accurately [9]. Their use of transfer learning also improved model accuracy, although the speed of identification remained a challenge, highlighting a need for further research into real-time applications. Moreover, in 2017, Huang et al. developed a fully convolutional network to identify water leakage in shield tunnels [10]. Their method, which classifies images into six categories, effectively mitigated interferences from elements such as tube sheet splices and pipelines, demonstrating strong robustness. Xue et al. in 2020 refined the Fast R-CNN framework by adjusting the scale parameters of anchor boxes, substantially optimizing the detection model [11].

Progress continued with Xiong et al. in 2020, who implemented a deep learning method to categorize water leakage images of shield tunnels, providing detailed characterizations for each category and validating the method’s efficacy against traditional approaches [12]. Gao et al. in 2019 developed an FRCNN (Fast Region Convolutional Neural Network) model that rapidly identified multiple tunnel diseases using the Faster R-CNN algorithm and an adaptive border ROI (region of interest) layer, addressing common interference issues and enhancing accuracy [13]. Liu et al. proposed an image enhancement algorithm applied to highway crack data to improve the accuracy of target disease detection and verified its effectiveness with three different target detection models [14]. Recently, in 2023, Zhou et al. introduced a YOLOv4 (You Only Live Once, Version 4)-based method for tunnel lining crack detection that employed a data enhancement technique to improve image quality and streamline the model for better detection efficiency and accuracy [15]. Additionally, in 2022, Kim et al. presented a six-step framework utilizing Cascade Mask R-CNN for non-cracked object tunnel crack detection, which achieved high accuracy and recall rates [16]. Lastly, Geng et al. in 2023 enhanced the Blend Mask image segmentation model, enabling precise disease extraction in tunnels and improving metrics such as recall, precision, dice coefficient, and mIoU (mean intersection over union), thus facilitating the quantification of tunnel diseases, although the presorting of disease-free images remains necessary [17]. Gu et al. in 2024 proposed the YOLOv8 dynamic plus model, which exhibited a 7.4 percentage-point performance improvement over the original YOLOv8 [18]. This enhancement effectively strengthens its capability in detecting building diseases. Han et al. in 2024 proposed an approach to construct a Res-Unet (U shape network) model by integrating the Unet model with the ResNet50 network, which ensures the accurate extraction of the entire fracture skeleton and the identification of minor cracks [19]. Lastly, Zhang et al. in 2025 proposed an enhanced YOLOv5 model with the ECA (Efficient Channel Attention) mechanism for automated GPR (Ground Penetrating Radar) defect detection, improving infrastructure health monitoring precision and efficiency and providing a robust solution for subsurface defect diagnosis in concrete structures like tunnel linings and bridge decks [20].

Current deep learning algorithms for identifying tunnel diseases predominantly focus on single disease targets and lack the capability to perform simultaneous multi-disease detection. In addition, while deep learning methods facilitate the detection and identification of tunnel diseases, they do not inherently quantify specific parameters of various disease types. This limitation necessitates the integration of image processing techniques and disease information extraction algorithms to enhance the detection, identification, and assessment of disease conditions.

To address these challenges, this paper proposes a multi-target rapid identification method for concrete structure diseases in tunnels. The proposed method innovatively combines the object detection network YOLOv7 with the segmentation network UNet, forming a Yolov7-UNet fusion model that achieves a closed loop of rapid recognition, precise segmentation and quantitative evaluation of diseases. Compared with traditional single-task models, the proposed approach realizes the simultaneous detection of multiple disease types including cracks, spalling, water leakage, and seam deformation. Furthermore, by integrating image processing techniques with disease information extraction algorithms, the proposed method can obtain quantitative parameters of detected defects, enabling comprehensive assessment and visualization of disease conditions. This approach enhances the efficiency, precision, and practicality of tunnel disease detection.

## 2. Multi-Disease Identification Network for Concrete Structures

### 2.1. Model Introduction

**Figure 1:**
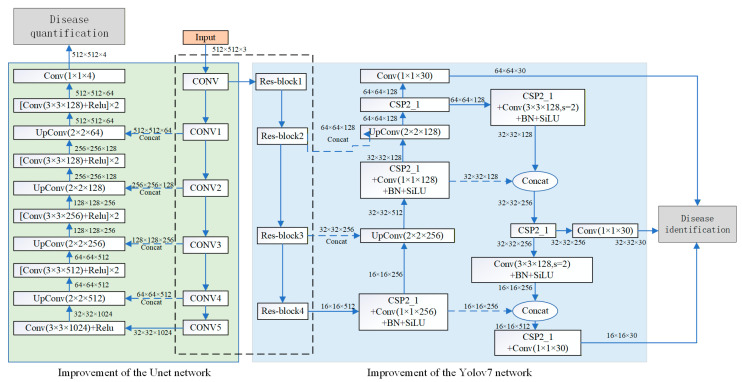
Multi-target identification and detection network structure.

In this paper, to address the challenge of detecting multiple visible diseases in tunnel lining concrete structures under specific environmental conditions, images captured by cameras are directly integrated into the computational framework. These annotated images are essential for training the two deep learning models, YOLOv7 and UNet. First, the YOLOv7-based object detection network identifies and classifies the diseases, providing preliminary localization. YOLOv7 was selected as the detection backbone because it offers an excellent trade-off between detection accuracy, inference speed, and computational efficiency. Although newer versions of YOLO have been proposed, YOLOv7 provides enough precision and robustness for the multitarget disease identification task in this study, while maintaining strong compatibility with the UNet segmentation network. This choice ensures model stability and efficiency without introducing unnecessary computational complexity. Then, the UNet-based semantic segmentation network precisely delineates and segments these diseases at the pixel level. To address challenges such as size imbalance and pixel conflict in the detection of multiple diseases, the traditional convolutional neural network is improved by employing specialized activation and loss functions. This refinement enables the network to screen images containing diseases efficiently, prioritize challenging samples during training, and predict the disease category at each pixel accurately. This approach not only facilitates precise segmentation of disease contours but also significantly accelerates detection process. After identifying the diseases, the segmented contours are quantified to extract detailed quantitative information about each disease. This comprehensive methodology is illustrated in the network structure presented in Figure 1.

The workflow begins with YOLOv7, which serves as the detection backbone. It receives the original and labeled images and generates bounding boxes for each identified disease. The YOLOv7 architecture consists of convolutional layers, residual connections, and multi-scale feature fusion, enabling the model to effectively capture both large and small disease features. After detection, the candidate regions generated by YOLOv7 are passed to the improved U-Net network for pixel-level segmentation. Detailed descriptions of the two improved networks are provided later in the text.

**Figure 2:**
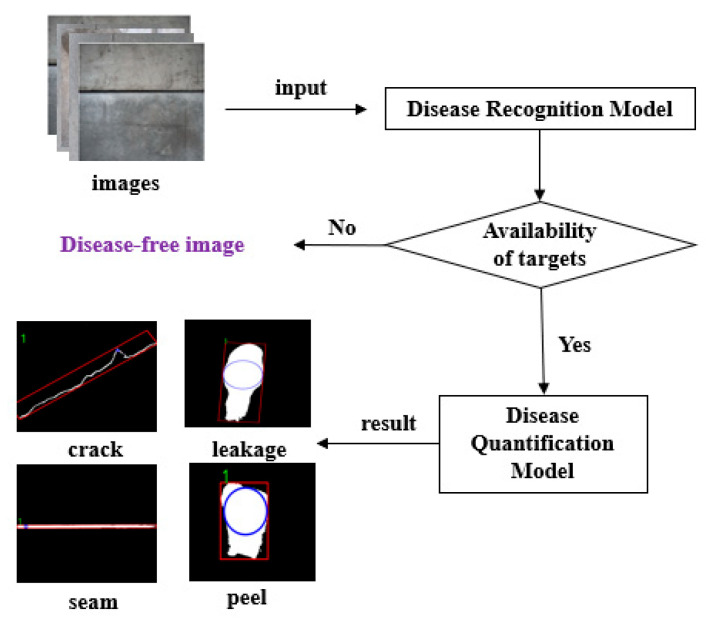
Multi-object identification and detection procedure.

As illustrated in Figure 2, the multi-target detection process begins with the standardization of input image, which is then fed into the detection network. If the image contains a disease, the network identifies the disease category, pinpoints its location, and calculates the confidence level. Conversely, if no disease is detected, the network classifies the image as a disease-free image, and the system analyzes the next image without additional action. Subsequently, for images identified with diseases, a specialized disease segmentation model is used to quantify and achieve fine pixel-level segmentation of the disease. This step allows for the precise extraction of the geometric dimensions and other critical parameters associated with each disease.

### 2.2. YOLOv7 Network

The network structure depicted in the blue section of Figure 1 is an improved YOLOv7 model, designed for the initial classification and localization identification of tunnel diseases. YOLOv7 is notable for its rapid detection capabilities; its base network achieves a frame rate of 45 fps, thereby meeting the requirements for real-time detection during routine tunnel inspections. The architecture is composed of four key components: input, backbone, Neck, and Prediction [21,22].

At the input stage, images are resized to a uniform dimension of 512 × 512 pixels before being passed to the detection network. In the Backbone module, the initial slicing operation is substituted with a stack of consecutive convolution layers with kernel 3 × 3, 6 × 6 and 3 × 3. This stack of convolutional layers increases the network depth, thereby strengthening its capacity to learn representative features. Feature extraction is facilitated by four residual blocks, each containing multiple residual units. Table 1 presents the specific convolutional feature layers and their corresponding output sizes in these residual blocks.

**Figure 3:**
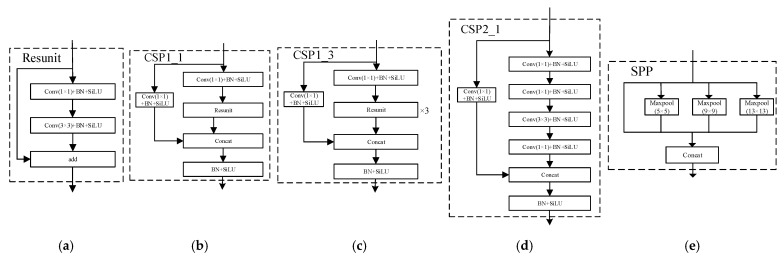
Residual cell flow diagram in an improved YOLOv7 structure. (**a**) Resunit block; (**b**) CSP1_1 block; (**c**) CSP1_2 block; (**d**) CSP2_1 block; (**e**) SPP block.

Figure 3 exhibits the flow of the residual units. The complete backbone network, denoted as “Backbone,” is composed of a cumulative total of 72 convolutional layers.

The Neck component adopts a fused Features Pyramid Network (FPK) and Path Aggregation Network (PAK) structure [23]. This architecture first propagates strong semantic features through a top–down pathway across multiple scales. Then, it conveys potent localization features in accordance through a bottom–up pathway. The fusion of these two pathways integrates feature maps from several detection layers in the backbone, thereby significantly enhancing the network’s feature capability.

In the Prediction component, multi-disease target classification is performed, where the YOLOv7 network is optimized using a binary cross-entropy loss function for each label. The binary cross-entropy loss function is tailored for binary classification tasks, and it is defined as follows:

(1) $$L\mathrm{oss}=-\frac{1}{N}y_{i}\cdot \log(p(y_{i}))+(1-y_{i})\cdot \log(1-p(y_{i}))$$

The regression loss function incorporates the *CIoU_Loss* regression method, which improves the speed and accuracy of prediction frame regression.

(2) $$CIOU\_LOSS=1-CIOU=1-(IOU-\frac{D\_2^{2}}{D\_C^{2}}-\frac{v^{2}}{(1-IOU)+v})$$

(3) $$v=\frac{4}{\pi^{2}}(\arctan\frac{w^{gt}}{h^{gt}}-\arctan\frac{w^{p}}{h^{p}})$$

where *y_i_* represents the label of the sample with positive class 1 and negative class 0, *p^i^* is the predicted probability for positive class, *D_2* is the Euclidean distance between the centers of the prediction box and the label box, *D_C* is the diagonal distance of the prediction box, *v* is a parameter that measures the consistency of the aspect ratio, *w^gt^* is the width of the label box, *h^gt^* is the height of the label box, *w^p^* is the width of the prediction box, and *h^p^* is the height of the prediction box.

### 2.3. UNet Network

The structure depicted in the green section of Figure 1 is the improved UNet model [24]. UNet is a foremost convolutional neural network utilized for image segmentation tasks, renowned for its efficacy in handling small targets or images with intricate details. UNet is specifically well-suited for detecting concrete cracks, seam deformation, and other similar pathologies. The UNet architecture consists of a symmetric encoder–decoder structure with four down-sampling stages and four up-sampling stages. The encoder comprises four identical down-sampling, each containing two 3 × 3 convolutional layers followed by a 2 × 2 pooling layer. The decoder similarly contains four up-sampling modules. Each module consists of a transposed convolutional layer for up-sampling, a feature concatenation layer, and two 3 × 3 convolutional layers. Table 2 presents the convolutional feature layer and output size of one of the down sampling modules. In the table, BN is short for Batch Normalization, while SiLU is short for Sigmoid Linear Unit and ReLU is short for Rectified Linear Unit.

First, a 512 × 512 image is input to the network, and padding is applied to its edges. Then, four consecutive down-sampling operations are executed. Each down-sampling module includes two convolutional layers and one pooling layer, ultimately generating 1024 feature maps with dimensions of 32 × 32. Similarly, four up-sampling operations are conducted, during which the feature maps are merged with the corresponding ones from the encoder via skip connections. Finally, a feature map of size 512 × 512 is obtained.

### 2.4. Quantification Method for Diseases

**Figure 4:**
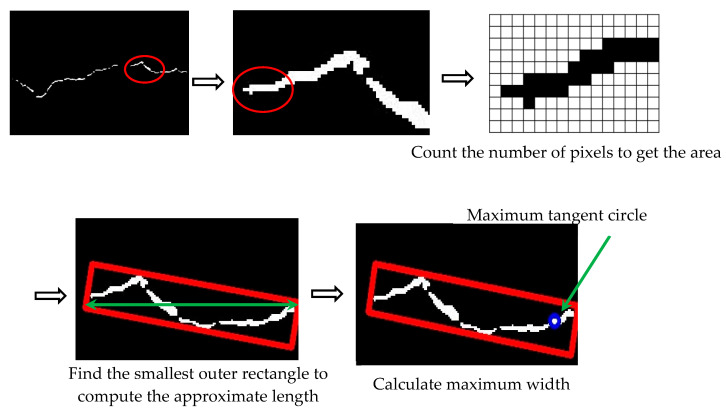
Measurement method of disease characteristic parameters.

Upon obtaining the binary image, numerous diseases within the original image can be effectively distinguished from the background area, presenting them as white region on a black background. Then, the disease parameters can be precisely identified and detected. Specifically, a minimum bounding rectangle is fitted to each segmented disease region. This step facilitates the measurement of key geometric attributes, including area, length, width, and other pertinent values. The specific detection steps are presented in Figure 4.

In this paper, a disease quantitative analysis algorithm is established to measure the feature parameters obtained from the semantically segmented binary image. The specific measurement methods for each feature parameter are described below.

(1) Length information

Length approximation involves constructing the minimum bounding rectangle enclosing the continuous binary image and calculating its diagonal length. 

Area is calculated by counting the total number of pixels within the continuous binary image and multiplying this count by the actual area represented by a single pixel. Width is calculated using two methods: The first method computes the diameter of maximum inscribed circle within the segmented defect region, representing the maximum width value of the disease. The second method employs the previously obtained length and area information and divides the area by the length to estimate the width value.

## 3. Fusion Deep Learning Network Training

### 3.1. Training Dataset

**Figure 5:**
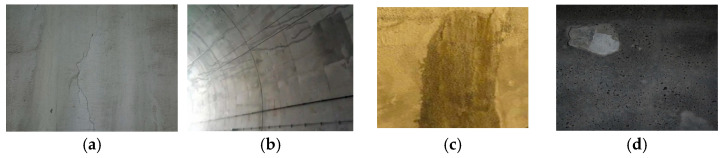
Typical disease maps in the dataset. (**a**) Crack; (**b**) seam; (**c**) water leakage; (**d**) spalling.

A small-scale dataset is selected to address the challenge of identifying tunnel-related diseases such as spalling, water leakage, cracks, and seam deformation. The dataset comprises 1022 images collected from multiple sources: actual tunnel image collections, snapshots from surveillance videos, inspection reports, photo libraries, and artificially generated sample. The dataset encompasses a variety of typical disease manifestations, as illustrated in Figure 5. Notably, some images contain multiple co-occurring disease types. To facilitate model training, the dataset is partitioned into a training set, a validation set and a test set in an 8:1:1 ratio. The specific categories and corresponding image counts are described in Table 3.

**Figure 6:**
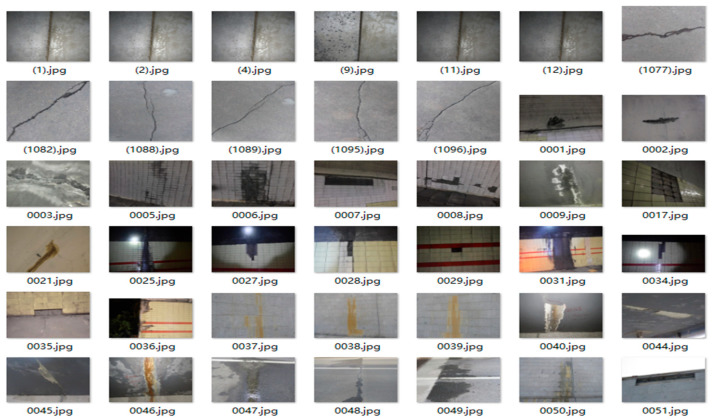
Typical disease figures from the training dataset.

To mitigate overfitting during the training phase and enhance the models’ overall performance and robustness, data augmentation techniques were applied to the dataset as shown in Figure 6. The primary augmentation techniques employed were Mosaic augmentation and random flipping. Mosaic augmentation randomly selects and crops four images from the dataset, then stitches them into a single composite image for training. This approach improves the batch size and reduces the computational cost per step, thereby improving training efficiency. Random flipping horizontally or vertically inverts the image data. This augmentation strategy expands the dataset while concurrently improving the model’s resilience to changes in input data. By incorporating these data augmentation techniques, the model improves its generalization capability, enhancing its performance and robustness on unseen data.

These two augmentation strategies were specifically chosen because they increase sample diversity without compromising the geometric integrity and thermal distribution characteristics that are critical for accurate defect recognition. Other augmentation techniques, such as color jittering, rotation, or scaling, were not employed, as they could distort the structural features of the tunnel lining, potentially misleading the model’s feature extraction. Therefore, the selected augmentation methods achieve a balance between data diversity and feature fidelity, enabling the model to generalize more effectively to unseen conditions while maintaining detection precision.

### 3.2. Network Evaluation Indicators

The YOLOv7 network employs the Intersection over Union (IoU) as an evaluation metric for anchor frame positioning. IoU is computed by comparing the intersection of the predicted and ground-truth bounding boxes with their union. A predefined IoU threshold is used as criterion to assign positive and negative samples during the training, Predictions with IoU above this threshold (typically set to 0.5) are considered positive samples, while those below are negative.

Precision and recall are key metrics for evaluating the accuracy of detection. Precision quantifies the fraction of correctly identified positive predictions among all positive predictions made by the model. It is defined as follows:

(4) $$P=\frac{TP}{TP+FP}$$

Recall measures the proportion of true positive samples correctly identified by the model. Recall is the classifier’s ability to detect all true positive samples during detection, and it is formally defined as follows:

(5) $$R=\frac{TP}{TP+FN}$$

The Average precision (*AP*) metric evaluates the overall performance of a trained model in detecting a specific object class across varying confidence thresholds. It is computed as the area under the Precision–Recall curve, which plots precision against recall at different classification thresholds. AP is determined as follows:

(6) $$AP=\sum_{i=1}^{n}P(R_{i})\Delta R_{i}$$

Mean average precision (mAP) is the average of the *AP* values across all detected categories. mAP is determined using Equation (7). Upon acquiring an ROI in a detected image, the target detection network usually applies the IoU threshold to the ROI against the ground truth bounding box. This step determines whether the ROI is classified as a positive or negative sample. The IoU threshold is a key hyper-parameter, typically set to 0.5, which remarkably affects the model’s detection accuracy. A lower threshold value means a higher model detection accuracy, whereas a higher threshold value implies a lower accuracy.

(7) $$mAP=\frac{\sum_{i=1}^{k}AP_{i}}{k}$$

The precision and recall metrics of the UNet network adhere to the same fundamental principles as those used in the YOLOv7 network. However, the comprehensive evaluation metrics are chosen from parameters commonly applied to semantic segmentation networks. In this paper, the comprehensive evaluation metric for the improved UNet network is the Dice coefficient, which quantifies the similarity between two sets on a scale of [0, 1]. A value of 0 indicates no overlap between the sets, while a value of 1 represents complete overlap and ideal segmentation performances. The Dice coefficient is defined as follows:

(8) $$Dice\;coefficient=\frac{2\times intersection\;area}{prediction\;area+real\;area}$$

The Dice coefficient is defined as twice the area of overlap between the predicted segmentation and the ground truth, divided by the sum of their individual. It yields a value of 1 for a perfect segmentation outcome.

### 3.3. Training Results

#### 3.3.1. YOLOv7 Model Training Results

**Figure 7:**
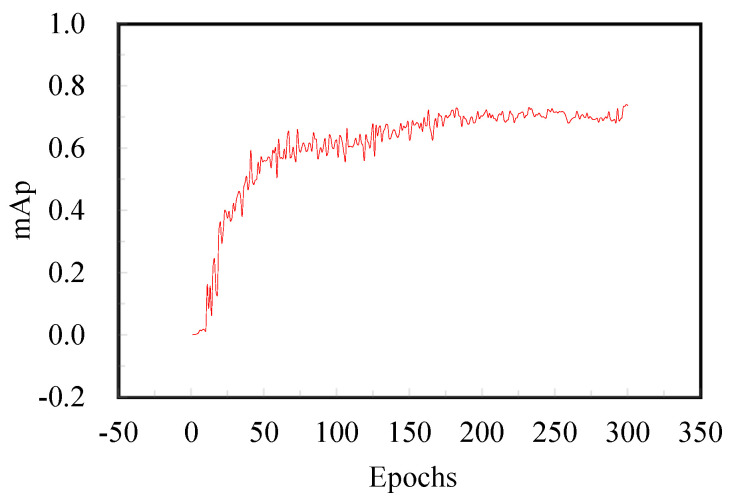
Object detection network training results.

In this paper, the enhanced YOLOv7 network is applied to the tunnel disease dataset with a batch size of 4. The specific results of the target detection network training are presented in Figure 7.

The model trained with the enhanced YOLOv7 network achieves an mAP of 0.75 after 300 epochs. This result indicates a high degree of overlap between the predicted bounding boxes and the ground-truth labels, demonstrating improved localization. Notably, this level of accuracy is maintained throughout the subsequent iterations of training, demonstrating stable model performance.

#### 3.3.2. UNet Model Training Results

**Figure 8:**
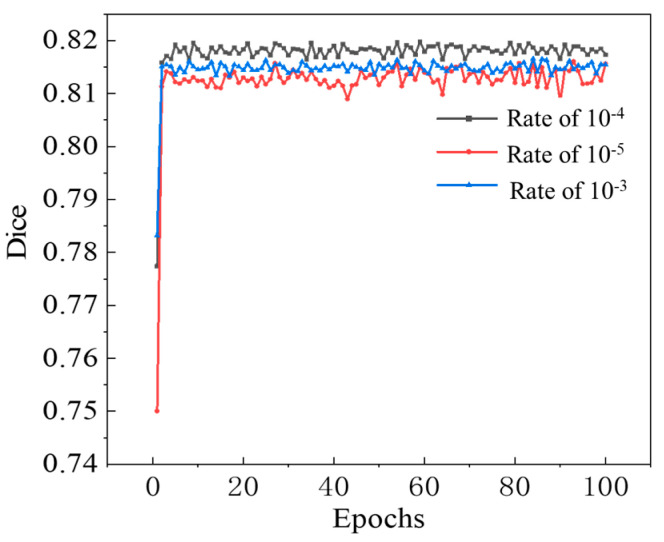
Network training results under different learning rates.

In this paper, an enhanced UNet network configured with 5 output categories and a batch size of 1 is selected. This section analyzes the impact of varying the learning rate parameter on the performance of the UNet network during both training and validation. We also investigate how the learning rate affects performance across different training dataset sizes. The experimental results are presented in Figure 8, which allows for a comparative evaluation of the network’s efficacy under various learning rate configurations.

The model achieved its optimal performance at the learning rate of 10^−4^. At this specific setting, the Dice coefficient reaches 0.82, revealing a high degree of similarity between the identification results and the ground truth. Consequently, this setting produces excellent identification results, demonstrating its effectiveness in accurately identifying target structures.

## 4. Disease Identification Tests

### 4.1. Overview of the Tests

**Figure 9:**
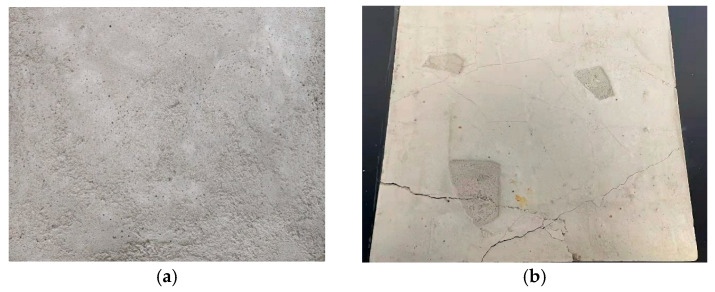
Concrete slab specimens. (**a**) intact specimen; (**b**) damaged specimen.

To improve the control of tunnel disease damage and guarantee accurate comparison and identification, we fabricated 12 concrete slabs to simulate various types of tunnel diseases. Each concrete slab measures 50 cm × 50 cm × 3.5 cm. A 4 mm diameter rebar was positioned within the casting template and secured in place with steel wires prior to concrete casting. Figure 9 illustrates the detailed production of these concrete slabs. Image processing techniques and quantitative modeling were then integrated to extract key parameters of the various diseases, enabling detailed analysis and evaluation.

The simulation of diseases encompasses numerous procedures to simulate real-world scenarios on concrete slabs. Hydraulic jacks were used to apply load to the slabs, creating cracks with varying widths ranging from 0.2 mm to 3 mm. Moreover, water was sprinkled onto the surface to simulate leakage conditions, and two slabs were joined to replicate common construction seams. Prior to complete curing, templates were inserted to build pits of differing shapes, mimicking spalling.

**Figure 10:**
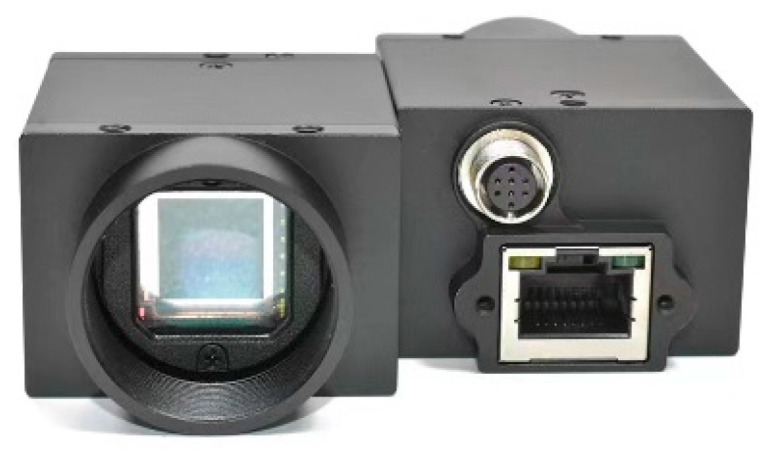
CMOS industrial camera.

**Figure 11:**
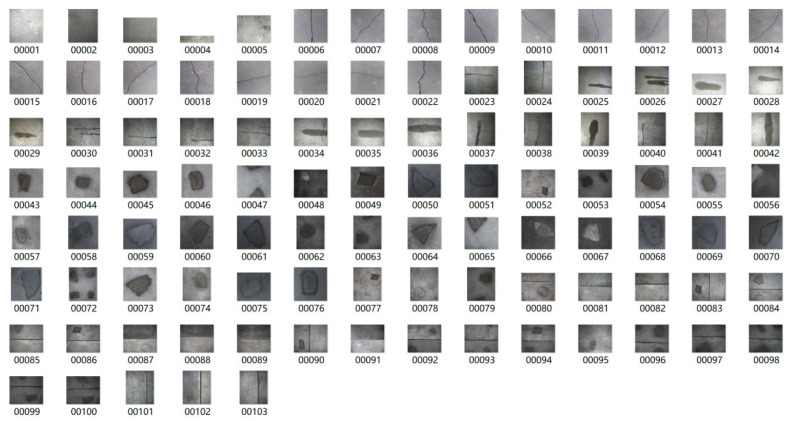
Disease image set.

Following fabricating concrete slaps with simulated diseases, images were captured using a CMOS industrial camera made by HIK VISION in Hangzhou of China. The key parameters of the camera are as follows: lens focal length is 16 mm; sensor size is 2/3 inch; pixel size is 3.45 μm × 3.45 μm; total number of pixels is 2448 × 2048; the maximum frame rate is 24 frames per second. These images maintain an elevated level of accuracy, with each pixel representing 0.1 mm. As shown in Figure 10, the images captured by the CMOS include 25 of cracks, 22 of water leakage, 37 of spalling, and 28 of seam diseases. Some images contain multiple co-occurring diseases, enhancing the comprehensiveness for analysis. Examples of these photographed images are presented in Figure 11.

### 4.2. Disease Identification Test Result

#### 4.2.1. Disease Target Detection Results

**Figure 12:**
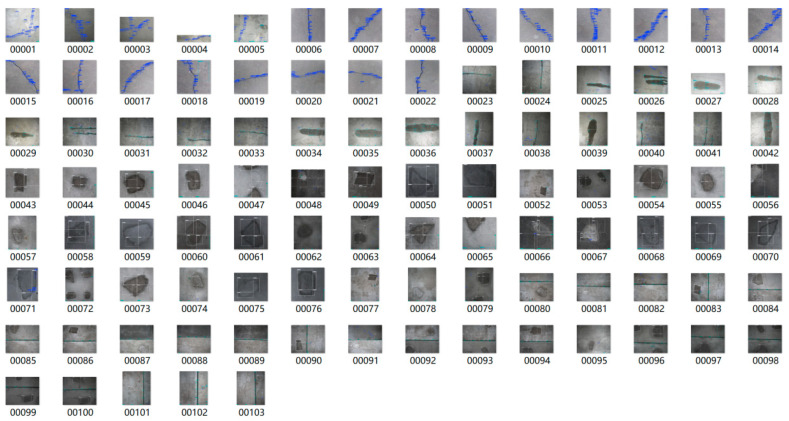
Typical prediction results of object detection model.

The custom-built concrete disease dataset was evaluated using the trained detection model. Figure 12 presents the detection results of the disease image set, and Table 4 provides a detailed quantitative summary.

**Figure 13:**
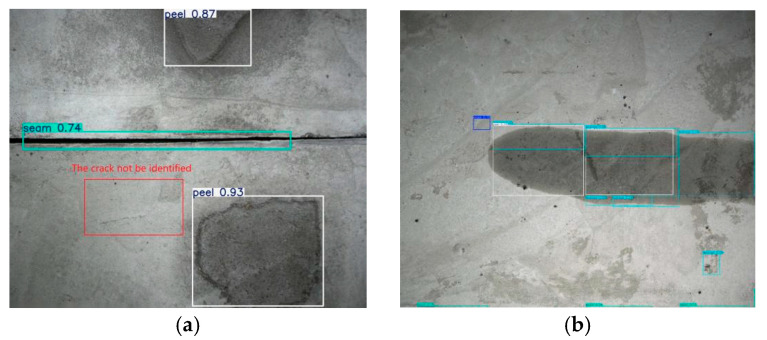
Cases of missed identification and misidentification. (**a**) Crack is not identified; (**b**) identified peel and leakage are not true peel and leakage.

Table 4 and Figure 12 show that the model correctly identifies most diseases, indicating its robust detection capability. However, some missed detection occurs, particularly for fine cracks less than 0.3 mm wide, as shown in Figure 13a. This outcome suggests the need for further investigation to strengthen the model’s ability to identify such fine features. Table 5 also shows numerous cases of misidentification, principally stemming from inherent pathologies in the dataset itself, such as anomalies in spalling. Misidentification refers to cases where a disease is detected in an image even though it does not actually exist. Missed identification refers to cases where a disease is present in the image but is not detected. Additionally, the frequent misidentification is largely due to dataset imperfections. For example, edges of spalling are occasionally misidentified as cracks or seams. Furthermore, the similarities between water leakage and spalling present challenges for accurate identification, as demonstrated in Figure 13b. Despite these challenges, the model exhibits initial proficiency in disease identification, providing a reliable foundation for subsequent segmentation and quantification of images. Furthermore, model refinement and dataset enhancement are essential to improve the model’s performance and effectively address these identification errors.

#### 4.2.2. Quantitative Analysis Results of Diseases

Following accurate disease detection by the YOLOv7-based target detection network, the identified disease regions in the images are segmented and quantified.

(1) Quantization results of crack identification

**Figure 14:**
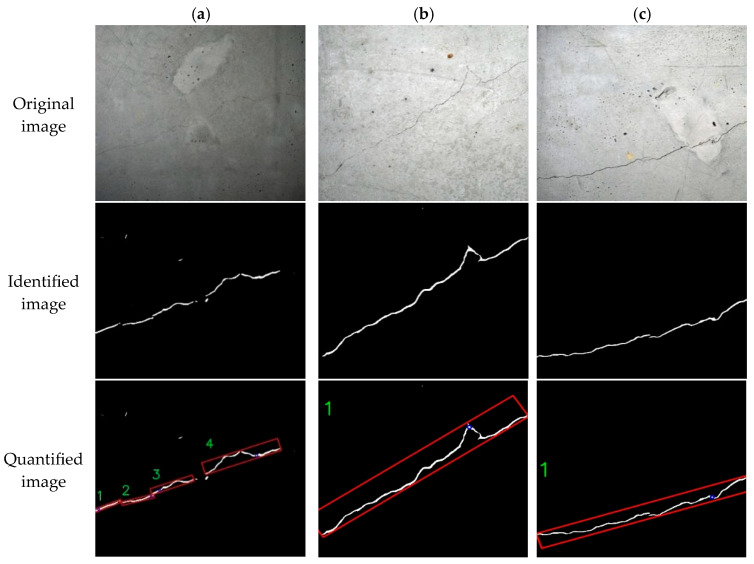
Quantitative identification test of crack information. (**a**) Image a; (**b**) Image b; (**c**) Image c.

The key geometric parameters measured for cracks are the maximum width and length [25]. The specific values for maximum width and length are determined by referencing Section 2.4. Figure 14 presents an example to demonstrates the accuracy of the crack parameter calculations.

Figure 14 shows three typical crack images processed by the UNet-based segmentation network to produce segmentation maps. Then, a quantitative analysis model measures the geometric parameters of the identified cracks. In Figure 14a, four crack regions are sequentially labeled and measured. The oriented minimum bounding rectangle surrounding each crack is highlighted by a red rectangle, and the maximum inscribed circle is denoted by a blue circle. These four labeled regions in fact form a single continuous crack. Hence, the maximum width of the entire crack can be determined by integrating the using the methodology for identifying multiple cracks through labeling with maximum inscribed circle algorithm. Subsequently, Figure 14b,c are identified as depicting a single continuous crack. The results of the quantitative identification of specific crack information are presented in Table 6 and Table 7.

The four regions identified in Figure 14a constitute a single crack, and the manual measurement of its widths are consistent with the quantitative calculation results from the semantic segmentation model, suggesting accurate identification. However, the initial width estimation method produced substantial calculation errors, resulting in undesirable outcomes. Hence, the maximum inscribe circle width method was adopted for all width calculation. Owing to segmentation errors, absolute errors range from 0.22 mm to 0.33 mm, with corresponding relative errors of 7.3–35.0%. Conversely, for the wider cracks in Figure 14b,c, both absolute and relative errors are significantly reduced, demonstrating better measurement accuracy. Measurement variability increases for narrower cracks, particularly those below 1 mm, due to the limited resolution where each pixel represents 0.1 mm. Thus, selecting a camera with higher spatial resolution would improve the measurement precision for fine crack.

(2) Quantitative results of seam identification

As width is a decisive parameter for seam identification, determining the maximum width of the seam using the maximum inscribe circle width method is more accurate.

**Figure 15:**
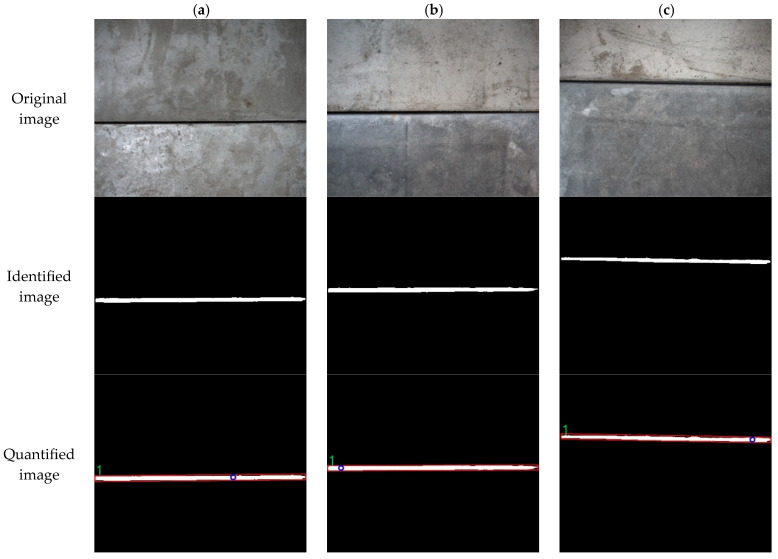
Quantitative identification test of seam information. (**a**) Image a; (**b**) Image b; (**c**) Image c.

In this paper, 12 concrete slabs were sequentially spliced to simulate tunnel seams, controlling the widths within the 1–4 mm range. A crack observer was used to measure the actual seam widths manually for subsequent accuracy assessment of quantitative calculations. Figure 15 exhibits the parameter information calculation for the test seams.

Figure 15 shows three typical seam images, processed using the same workflow as for the crack tests. The measured seam widths are 2.60 mm, 2.50 mm, and 2.50 mm, for Figure 15a–c, respectively. The seam widths are wider than the cracks. According to relevant tunnel specifications, 4 mm serves as a critical value for seam width. The results of quantitative identification for specific seam information are summarized in Table 8.

The seams display a regular, predominantly straight shape, and the measured lengths agree well with the lengths calculated by the algorithm. When the actual width is over 1 mm, the calculation results from quantitative identification produce small absolute errors. The widths determined using the maximum inscribe circle width method closely approximate the actual values, with relative errors under 10%. These findings further emphasize the effect of spatial resolution on the precision of disease detection and identification.

(3) Quantitative results of spalling identification

Detection and identification of spalling diseases are relatively straightforward, with area serving as the primary parameters for quantification.

**Figure 16:**
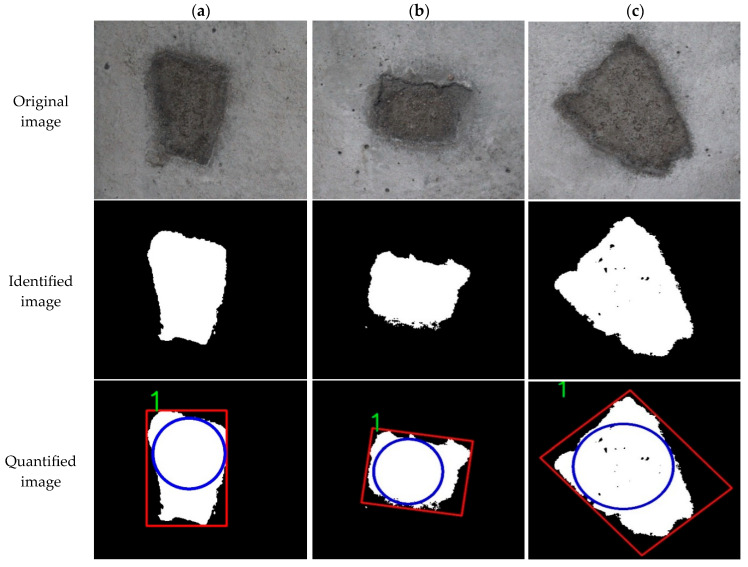
Quantitative identification test of spalling information. (**a**) Image a; (**b**) Image b; (**c**) Image c.

Figure 16 shows three typical images depicting spalling diseases. Compared to cracks and seams, spalling in tunnel environments usually covers larger areas. With the unit pixel accuracy remaining consistent at 0.1 mm/pixel, the system guarantees adequate accuracy for detecting and identifying spalling damage. The quantification results for the spalling information are summarized in Table 9.

The number of pixels occupied by the spalling disease substantially exceeds that of cracks, seams, and other diseases, which facilitated more reliable detection due to their larger spatial extent. The area of the spalling, determined based on the number of pixels, closely aligns with the measured area, meeting the detection requirements effectively.

(4) Quantitative results of water leakage identification

The water leakage area serves as the core index for quantitative identification. To perform the quantitative identification test of water leakage information, concrete slabs were mounted against a wall and simulated to develop water leakage. The local length and width of the water leakage were then determined with a tape measure.

**Figure 17:**
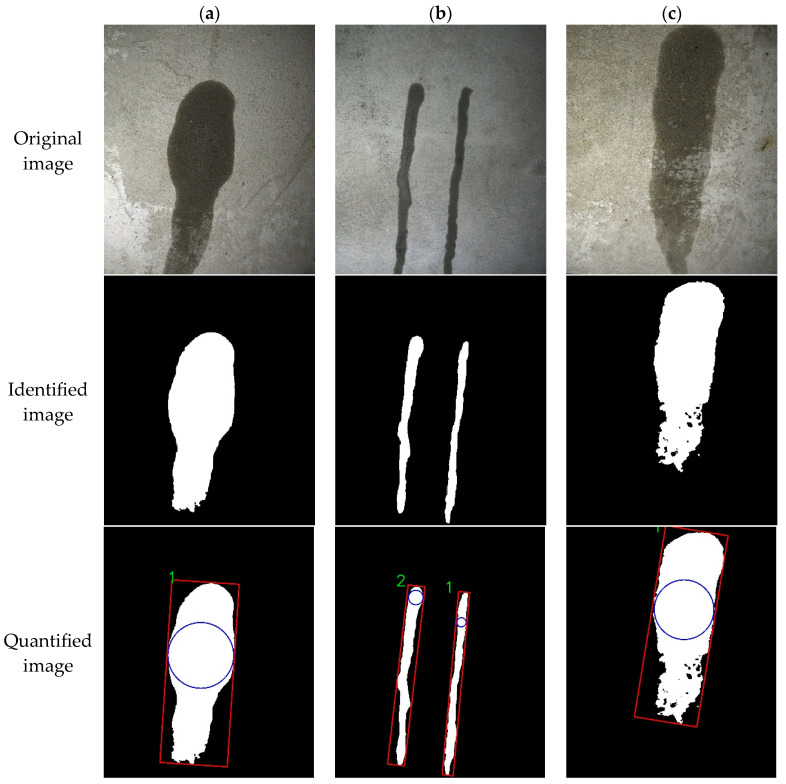
Quantitative identification test of leakage water information. (**a**) Image a; (**b**) Image b; (**c**) Image c.

Figure 17 illustrates three typical images containing water leakage, and the water leakage region occupies a considerable part of the entire image. The results of quantitatively identifying specific water leakage information are shown in Figure 17.

Similar to the spalling diseases the water leakage diseases observed in the tunnel typically cover large areas. Consequently, the overall size occupies a larger number of pixels at the spatial resolution of 0.1 mm/pixel. This unit pixel accuracy is sufficient for accurately detecting and identifying water leakage diseases. The results of quantitative results for water leakage information are shown in Table 10.

The quantification of water leakage diseases involves parameters such as length and area. Their characteristic shapes, typically narrow vertical lines or spindle forms that occupy a lot of pixels, contribute to dependable detection accuracy. As shown in Figure 17b, the quantification identification algorithm presented in this paper can sequentially quantify and identify multiple instances of water leakage within a single image. This advantage enables comprehensive detection of multi-disease targets within an image, maximizing detection capability.

(5) Quantitative results of multi-disease identification

**Figure 18:**
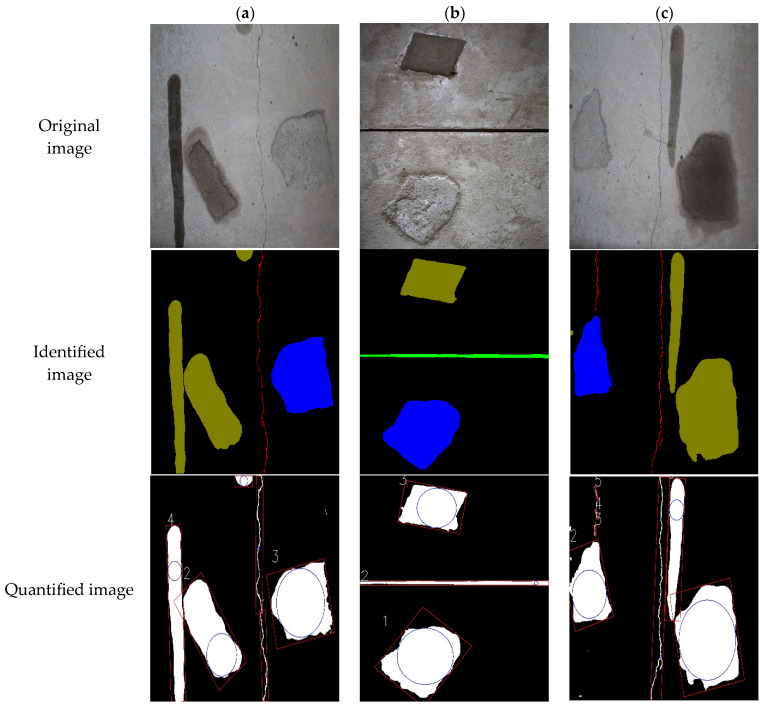
Quantitative identification test of multiple diseases. (**a**) Image a; (**b**) Image b; (**c**) Image c.

In the daily inspection of tunnels, encountering areas with multiple tunnel structure diseases is common. To assess the quantitative identification of multi-disease information on tunnel concrete lining, fabricated concrete slabs were mounted against the wall to simulate scenarios with multiple diseases in this paper. The same methodology described previously was applied to identify and quantify the corresponding key parameters of multiple diseases. The results of the quantitative identification of specific water leakage information are shown in Figure 18.

Figure 18 displays three images with multiple diseases. The network trained in this paper successfully identifies images containing multiple diseases and distinguishes between several types of diseases by assigning different color regions, namely, red for cracks, blue for spalling, olive for water leakage, and green for seams. Subsequently, the proposed feature measurement methods are used for quantification, and the specific quantitative identification results for multi-disease information are presented in Table 11 and Table 12.

In this paper, the quantified information of multiple diseases in images is calculated separately. The experimental results of the quantitative identification of multiple diseases reveal that the proposal fusion network model can effectively identify multiple diseases within a single picture, with an accuracy comparable to that achieve for single diseases mentioned previously. The identification accuracy for larger diseases such as spalling and water leakage meets detection requirements, with relative errors below 10%. However, for cracks and seams, the identification error is influenced by the actual width. While wider cracks show relative errors below 12%, narrow ones exhibit larger relative errors.

Overall, the quantitative identification method of tunnel damage based on deep learning effectively extracts various disease features. Based on the connected component analysis, the calculation of parameter information for various diseases exhibits a certain degree of reliability. However, accurate quantitative identification of fine cracks requires high-performance imaging hardware to ensure result reliability.

#### 4.2.3. Disease Identification Efficiency Tests and Results

This section introduces the concrete multi-disease identification fusion network, which primarily aims at improving the speed and efficiency of tunnel detection. To assess its effectiveness, 40 images of custom-fabricated concrete blocks containing diseases and 40 images without diseases, totaling 80 images, were selected as the detection samples. These sample datasets were then subjected to detection using the UNet network and the concrete structural disease multi-objective identification network proposed in this paper. The detection times of both methods were then compared. The experimental comparison results are summarized in Table 13.

Integrating a YOLOv7-based detection network into the disease screening removes the need to segment and quantify disease-free images. This integration significantly reduces the detection time, with the fusion network completing the task in less than half the time required by the UNet network alone. Consequently, the overall detection speed substantially improves.

Because tunnels currently in operation are generally in sound condition, the number of documented disease cases available for study is limited. Consequently, the number of images containing diseases in the collected dataset is small. In such scenarios, a detection method that first screens and classifies all collected images before segmenting identified diseases can effectively accelerate the entire detection. This approach optimizes efficiency by concentrating computational resources on images with a high probability of containing diseases, thereby streamlining the detection and improving overall throughput.

## 5. Conclusions

Based on deep learning networks, this paper introduces a multi-objective identification framework for tunnel diseases through a novel YOLO–UNet fusion model. The following conclusions are drawn from the indoor simulation experiment conducted on custom-fabricated concrete slabs:

(1) The proposed fusion network demonstrates robustness in identifying and segmenting diseases, achieving an mAP of 0.75 and a Dice score of 0.82 on the training dataset.
(2) The proposed multi-objective identification network model remarkably improves the identification speed compared with the UNet network model. The proposed fusion network can identify the diseases about 50% faster than UNet.
(3) The fusion network model presented in this paper has satisfactory recognition accuracy. The original diseases in the test images are successfully recognized, with only a few minor cracks being missed.
(4) The proposed method achieves quantitative assessment of multiple tunnel diseases, with relative errors below 11.07% for the area of water leakages, below 5% for the length and area of cracks, and below 6% for the width of seams, thus realizing a closed-loop process of rapid recognition–precise segmentation–quantitative evaluation.

In terms of the detection speed, identification accuracy, and quantization index accuracy, the method proposed in this paper shows substantial value for practical engineering applications. However, shortcomings remain. The fusion network model requires 0.35 s to process a single disease map, leaving room for optimization.

Future research will focus on developing more lightweight and efficient fusion networks to achieve real-time disease detection and seamless deployment in practical tunnel monitoring systems. In addition, introducing multimodal sensing data, such as infrared thermal imaging, laser scanning, and acoustic sensing, could enhance the detection accuracy and robustness of the model under complex environmental conditions. Additionally, for crack width quantification, the error tends to increase for finer cracks. This limitation may be mitigated by using higher-resolution imaging equipment or by integrating more precise quantification algorithms. Lastly, the model generalization capability should be verified in the field application.

## Figures and Tables

**Table 1:** Residual block convolutional feature layer and output size.

Layer	Convolutional Feature Layer	Output Size
CONV	Conv(3 × 3 × 64) + BN + SiLU	512 × 512 × 64
Res-block1	Conv(6 × 6 × 64, s = 2) + BN + SiLU	128 × 128 × 64
	Conv(3 × 3 × 64, s = 2) + BN + SiLU	
	CSP1_1	
Res-block2	Conv(3 × 3 × 128, s = 2) + BN + SiLU	64 × 64 × 128
	CSP1_3	
Res-block3	Conv(3 × 3 × 256, s = 2) + BN + SiLU	32 × 32 × 256
	CSP1_3	
Res-block4	Conv(3 × 3 × 256, s = 2) + BN + SiLU	16 × 16 × 512
	SPP	

**Table 2:** Subsampled convolution feature layer and output size.

Layer	Convolutional Layer Feature	Output Size
CONV	Conv(3 × 3 × 64) + BN+ SiLU	512 × 512 × 64
CONV1	Conv(3 × 3 × 64) + BN + ReLU	512 × 512 × 64
CONV2	Maxpool(3 × 3)	256 × 256 × 128
	Conv(3 × 3 × 128) + BN + ReLU	
	Conv(3 × 3 × 128) + BN + ReLU	
CONV3	Maxpool(3 × 3)	128 × 128 × 256
	Conv(3 × 3 × 256) + BN + ReLU	
	Conv(3 × 3 × 256) + BN + ReLU	
CONV4	Maxpool(3 × 3)	64 × 64 × 512
	Conv(3 × 3 × 512) + BN + ReLU	
	Conv(3 × 3 × 512) + BN + ReLU	
CONV5	Maxpool(3 × 3)	32 × 32 × 1024
	Conv(3 × 3 × 1024) + BN + ReLU	

**Table 3:** Category and number of images.

Numbers	Classes	Numbers
1	crack	180
2	water leakage	84
3	spalling	308
4	seam	450

**Table 4:** Detection result.

Classes	Number of Images	Correctly Identified	Identification Rate
Crack	25	22	88%
leakage	22	22	100%
peel	37	37	100%
seam	28	28	100%

**Table 5:** Number of misidentification and missed identification.

Classes	Misidentifications	Missed Identifications
crack	7	3
leakage	10	0
peel	14	0
seam	7	0

**Table 6:** Quantitative identification results of crack length information.

Image	Classes	Label	Calculated Length /mm	Measured Length/mm	Absolute Error /mm	Relative Error
a	crack	1	34.49	34	0.49	1.44%
a	crack	2	40.89	41	0.11	0.27%
a	crack	3	56.44	58.9	2.56	4.35%
a	crack	4	102.04	107	4.96	4.64%
b	crack	1	198.41	209	10.59	5.07%
c	crack	1	152.73	161	8.27	5.14%

**Table 7:** Quantitative identification results of crack width information.

Image	Classes	Label	Maximum Inner Circle Width/mm	Measured Width/mm	Absolute Error/mm	Relative Error
a	crack	1	1.08	0.80	0.28	35.0%
a	crack	2	1.02	0.80	0.22	27.5%
a	crack	3	1.03	0.80	0.23	28.8%
a	crack	4	1.13	0.80	0.33	41.3%
b	crack	1	1.49	1.30	0.19	14.6%
c	crack	1	1.18	1.10	0.08	7.3%

**Table 8:** Quantitative identification test of seam information.

Image	Classes	Label	Maximum Inner Circle Width/mm	Measured Width/mm	Absolute Error/mm	Relative Error
a	seam	1	2.75	2.60	0.15	5.8%
b	seam	1	2.65	2.50	0.15	6.0%
c	seam	1	2.61	2.50	0.11	4.4%

**Table 9:** Quantitative identification test of spalling information.

Image	Classes	Label	Pixel Number	Area/mm^2^	Measured Area/mm^2^	Absolute Error/mm^2^	Relative Error
a	spalling	1	214,588	2145.88	2079	66.88	3.2%
b	spalling	1	209,437	2094.37	2026	68.37	3.4%
c	spalling	1	463,126	4631.26	4427	204.26	4.6%

**Table 10:** Quantitative identification test of leakage water information.

Image	Classes	Label	Area/mm^2^	Measured Area/mm^2^	Absolute Area	Relative Area
a	water leakage	1	8165.9	8376	210.6	2.5%
b	water leakage	1	1291.99	1376	84.01	6.1%
b	water leakage	2	1628.41	1775	146.59	8.3%
c	water leakage	1	8726.07	9882	1155.93	11.7%

**Table 11:** Quantitative identification test of cracks and seam.

Image	Label	Classes	Calculated Length/mm	Measured Length/mm	Relative Error	Maximum Inner Circle Width/mm	Measured Length/mm	Relative Error
a	1	crack	83.69	92	9.0%	1.02	1.1	7.3%
a	5	crack	125.87	125	0.7%	1.12	1.1	1.8%
c	3	crack	10.65	10	6.5%	0.32	0.2	60.0%
c	4	crack	11.27	9	25.2%	0.42	0.3	40.0%
c	5	crack	13.82	10	38.2%	0.27	0.2	35.0%
c	7	crack	207.06	218	5.0%	1.12	1	12.0%
b	2	seam	204.77	207	1.1%	2.05	2.1	2.4%

**Table 12:** Quantitative identification test of water leakage and spalling.

Image	Label	Classes	Area/mm^2^	Measured Area/mm^2^	Absolute Error	Relative Error
a	3	spalling	5378.98	5550	171.02	3.1%
b	1	spalling	4424.77	4548	123.23	2.7%
c	2	spalling	2636.22	2986	349.78	11.7%
a	2	leakage	3550.12	3240	310.12	9.6%
a	4	leakage	2431.47	2516	84.53	3.4%
a	6	leakage	175.38	192	16.62	8.7%
b	3	leakage	2552.41	2824	271.59	9.6%
c	1	leakage	6014	5892	122	2.1%
c	6	leakage	1781.87	1856	74.13	4.0%

**Table 13:** Several network detection operation results.

Network Model	Number of Images	Time
UNet	80	29.759 s
Method proposed in this paper	80	14.139 s

## Data Availability

No research data available.
